# Perspectives on the Search for a True Physiologic Replacement Therapy for Hypoparathyroidism

**DOI:** 10.17925/EE.2016.12.01.47

**Published:** 2016-03-15

**Authors:** Karen K Winer

**Affiliations:** NICHD, National Institutes of Health, Bethesda, Maryland, US

**Keywords:** Parathyroid hormone (PTH) 1-34, calcium metabolism, PTH analogs, APS-1, calcium receptor, vitamin D

## Abstract

Over the past two decades, we have studied various parathyroid hormone (PTH) 1-34 regimens, including once-daily and twice-daily injections without the concurrent conventional therapy. We recently studied PTH delivery by insulin pump, which produced normal, steady-state serum and urine calcium levels. The recent approval of PTH 1-84 represents an important milestone in the treatment of hypoparathyroidism. As PTH 1-84 and PTH 1-34 have similar pharmacokinetic (PK) and pharmacodynamic profiles, one can assume that many of the principles learned from studies of PTH 1-34 also apply to PTH 1-84 in the management of this rare disease.

Hypoparathyroidism is a rare disease, characterised by low or undetectable serum parathyroid hormone (PTH), hypocalcemia, hyperphosphatemia and, frequently, hypomagnesaemia. Symptoms include neuromuscular irritability causing tetany, muscle cramping and seizures. In adults, the disorder is usually a complication of neck surgery. In children, the condition is most often due to inherited disorders such as autoimmune polyglandular failure syndrome type 1 (APS-1) or an activating mutation in the calcium-sensing receptor (CaR).

PTH is the key endocrine regulator of calcium homeostasis. The principle target organs for PTH action are the kidney and the bone. PTH has indirect effects on the intestinal tract through the stimulation of 1,25(OH)_2_ vitamin D production in the kidney. The calcium receptor, found in many areas of the body, partially controls calcium excretion in the kidney and PTH secretion from the parathyroid gland.

Conventional therapy consists of multiple oral doses of vitamin D analogues and calcium. Although this therapy is effective in raising serum calcium, it does not fully restore normal mineral homeostasis. It bypasses the PTH effects on the kidney and bone and relies entirely on calcium transport across the gastrointestinal tract to normalise blood calcium. Without the renal calcium-retaining effects of PTH, conventional therapy may lead to renal insufficiency due to progressive nephrocalcinosis.

Until recently, the need for a replacement therapy for hypoparathyroidism had been largely ignored by the endocrine community. Although PTH first became available nearly a century ago, no controlled studies investigating replacement therapy took place before 1990. Ninety years ago, Collip demonstrated that bovine PTH was an effective replacement therapy in parathyroidectomised dogs.^1^ In 1925, Albright treated a 14-year old boy with bovine PTH.^2^ However, once vitamin D became the treatment of choice for hypoparathyroidism, Albright did not further pursue studies of PTH therapy. Investigation into PTH replacement remained inactive for the next 60 years, until 1990.

In the 1970s, the sequencing of the biologically active fragment, PTH 1-34, led to the study of its physiological effects, which revealed its dose-dependent anabolic effects on bone. The development of PTH 1-34 as an anabolic agent for osteoporosis continued through the next three decades and culminated in 2002 with the approval of teriparatide (rhPTH 1-34) (Forteo®, Lilly, Indianapolis, US) for osteoporosis.^3^

In 1990, Strogmann reported the successful treatment of two adolescents with hypoparathyroidism with PTH 1–38 injections.^4^ In 1991, we initiated the first randomised controlled studies of replacement therapy with synthetic human PTH 1–34.^5^ The majority of patients referred to us for therapy had evidence of renal calcifications. Some of these patients had renal insufficiency and others were in renal failure. These comorbidities were a direct result of conventional therapy or recurrent intravenous calcium infusions to treat intermittent severe hypocalcemia.

Over the past 25 years, we have evaluated various dose regimens of synthetic human PTH 1–34, including once-daily and twice-daily subcutaneous PTH 1–34 injections without the use of calcitriol or calcium supplements in adults and children of all aetiologies. The National Institutes of Health (NIH) pharmacy formulated synthetic human PTH 1–34 in single dose vials. The PTH dosage was individually titrated to achieve optimal calcium homeostasis. Increased frequency of injections was associated with a significantly reduced total daily dose of PTH.^6^ A three-year controlled study comparing PTH to conventional therapy in both adults and children confirmed that PTH could maintain normal serum and urine calcium levels and was an effective long-term therapy. Bone density remained stable in adults and children had normal bone accrual.^7,8^ To further refine replacement therapy, we studied PTH delivery by insulin pump. A significant therapeutic breakthrough in the study of hypoparathyroidism, pump delivery produced normal, steady-state urine and serum calcium levels. PTH delivered by pump allowed for simultaneous normalisation of bone markers, serum calcium, magnesium and urine calcium excretion levels in patients of all aetiologies, including congenital hypoparathyroidism.^9,10^

We have arrived at three essential therapeutic principles that underlie successful treatment of this rare disorder.

- Individualised PTH requirements depend upon the disease aetiology.
- Smaller, more frequent doses of PTH replacement by subcutaneous injection reduce stimulation to bone and kidney and result in lower calcium excretion and markers of bone turnover. Most outcome measures are dose dependent, including blood and urine calcium levels, markers of bone turnover, bone density in adults and bone accrual in children.
- PTH delivered by pump produced the most physiological biochemical profile.

PTH 1–84 was recently approved by the US Food and Drug Administration for the treatment of hypoparathyroidism as an adjunct to conventional therapy in patients who are refractory to conventional therapy alone. Although PTH 1–34 and PTH 1–84 likely have identical biological effects, studies of these two peptides represent divergent approaches to replacement therapy, which has led to different treatment guidelines. PTH 1–34 has been given, from its very early investigative stages, as multiple subcutaneous injections titrated individually to normalise both blood and urine calcium levels. PTH 1–84, on the other hand, has been given as a fixed daily dose or on alternate days, along with flexible dosing of conventional therapy. This approach ignores a key principle that has emerged through our work: the PTH dose size and frequency has a remarkable effect on metabolic control. Claims that the longer half-life of PTH 1–84 drives the dosing requirements remain unsupported. Although there are no data comparing the two peptides, separate pharmacokinetic (PK) studies demonstrate that they are very similar.^11,12^

Study results with fixed daily PTH 1–84 doses raise questions regarding the potential long-term effects of this approach. Sikjaer et al., showed that 100 mcg of PTH 1–84 given once daily along with conventional therapy produced hypercalcemia six to 14 hours after the injection.^12^ The recent multicentre Randomised, Double-blind, Placebo-controlled, Phase 3 Study to Investigate the Use of NPSP558, a Recombinant Human Parathyroid Hormone (rhPTH[1–84]) for the Treatment of Adults with Hypoparathyroidism (REPLACE) study reported higher mean urine calcium levels in the treatment group than in the placebo group.^13^ Longterm treatment studies report both anabolic^14,15^ and catabolic effects^16^ on bone density in adult patients. These challenges could be overcome with more focused titration of the PTH 1–84 dose based on individual needs.

Many cases of hypoparathyroidism are refractory to conventional therapy. This includes patients with chronic hypercalciuria with either nephrocalcinosis or evidence of decreased renal function. Some patients with APS-1 have malabsorption and are calcium infusion dependent. Off-label PTH treatment remains a vital option for these patients. Twice-daily injections of Natpara® (parathyroid hormone; Shire, US) individually titrated to normalise serum and urine calcium levels without concurrent calcitriol and calcium would be considered off-label use of this relatively new drug. Many doctors have prescribed rhPTH 1–34 (Forteo) off label, for treatment of their patients with hypoparathyroidism, since its approval in 2002. After completing our studies at the NIH, many protocol subjects have turned to Forteo, which is dispensed in a pen injector. The 20 mcg daily dose approved for osteoporosis may cause transient elevations in blood and urine calcium in most patients with hypoparathyroidism. Patients therefore are often instructed to use the pen injector as a multidose vial and withdraw the prescribed amount of PTH using an insulin syringe. Patients typically start on a 10 mcg twice-daily dose and are titrated from that point.

Future directions include the need for long-term studies with pump delivery of PTH 1–34. The comparison of PTH 1–84 and PTH 1–34 may elucidate differences between these two peptides. PTH 1–84 should be further studied as a true replacement therapy, titrated to the individual needs of the patient, without concurrent treatment with calcitriol and calcium. Technological advances in monitoring have revolutionised diabetes management and could have a similar impact on hypoparathyroidism to help mitigate against episodes of hypocalcemia. To take the diabetes analogy further, management of that disease has relied heavily on the concurrent use of insulins with different PK profiles. Similarly, the use of longer-acting peptides may further reduce calcemic variability and become the new frontier in the management of hypoparathyroidism.
